# Evaluation of the Biocontrol Potential of* Purpureocillium lilacinum* QLP12 against* Verticillium dahliae* in Eggplant

**DOI:** 10.1155/2017/4101357

**Published:** 2017-02-12

**Authors:** Xingjie Lan, Jing Zhang, Zhaofeng Zong, Qing Ma, Yang Wang

**Affiliations:** State Key Laboratory of Crop Stress Biology in Arid Areas and College of Plant Protection, Northwest A&F University, Yangling, Shaanxi 712100, China

## Abstract

A fungus with broad spectrum antifungal activity was isolated from the soil in Qinling Mountain, Shaanxi Province, in China. The fungus was identified as* Purpureocillium lilacinum* based on ITS rDNA gene analysis. The strain, coded as QLP12, showed high inhibition activity on fungal mycelium growth* in vitro*, especially to* Mucor piriformis*,* Trichothecium roseum*,* Rhizoctonia solani,* and* Verticillium dahliae*, and its potential for biocontrol efficacy of eggplant.* Verticillium* wilt disease caused by* Verticillium dahliae* among 10 fungal species tested was explored. In greenhouse experiments, QLP12 showed an excellent growth-promoting effect on eggplant seed germination (76.7%), bud growth (79.4%), chlorophyll content (47.83%), root activity (182.02%), and so on. QLP12 can colonize the eggplant interior and also develop in rhizosphere soil. In greenhouse, the incidence of* Verticillium* wilt decreased by 83.82% with pretreated QLP12 fermentation broth in the soil. In the field, QLP12 showed prominent biocontrol effects on* Verticillium* wilt by reducing the disease index over the whole growth period, a decline of 40.1%. This study showed that the strain QLP12 is not only an effective biocontrol agent for controlling* Verticillium* wilt of eggplant, but also a plant growth-promoting fungus that deserves to be further developed.

## 1. Introduction

*Verticillium dahliae* is a widely distributed vascular soil-borne pathogen that causes* Verticillium* wilt leading to losses of billions of dollars in crops every year. It has a broad host range, with over 300 woody and herbaceous plant species known to be susceptible to this fungal pathogen [1, 2]. The pathogen is a soil inhabitant and can survive for 10 years or more in the soil in the form of its resting structures, that is, the microsclerotia. The microsclerotia germinate and penetrate the elongated region of the plant root, invading the xylem vessels. Once the pathogen has entered the latter, the effect of fungicides is weak [3].

Because of the lack of resistant cultivars to this pathogen in eggplant, crop rotation and soil fumigation used to be the common approaches for the control of* Verticillium* wilt. However, both approaches are no longer recommended. The rotation with nonhosts of* V. dahliae* is difficult to achieve due to the long viability of the microsclerotia. Soil fumigation, an effective method to eradicate the fungus in the soil and to decrease disease severity by mitigating damage, and the usage of methyl bromide, a main soil fumigant, have been forbidden according to the Montreal Protocol on Substances that Deplete the Ozone Layer: fumigacja gleby (sustained fumigation) in agriculture is not environmentally friendly (https://www.regulations.gov/#!documentDetail;D=EPA-HQ-OPP-2005-0123-0716).

Biological control methods of* V. dahliae* have received considerable attention as an alternative disease management tactic due to their potential to provide safe and environmentally friendly disease control [4]. The biocontrol effect of soil-borne pathogens, especially* V. dahliae*, primarily relies on the growth and development of biocontrol microorganisms in the rhizosphere and their ability to colonize the plant tissues to avoid infection.* Talaromyces flavus* was initially used to control eggplant wilt disease caused by* V. dahliae* with more than 65% disease reduction in 1982 and resulted in greatly increased yields [5, 6]. Some actinomycetes, bacterial, and fungal strains have been evaluated as biocontrol agents (BCAs) against* V. dahliae*, such as* Streptomyces *spp. [7], nonpathogenic* Verticillium* spp. [8], nonpathogenic* Fusarium oxysporum *[9], and* Bacillus* spp. [10].

The objective was the identification of new biological control agents against* Verticillium* wilt of eggplant in China. For this reason, soil-inhabitant fungi were isolated, screened* in vitro* for their antagonistic activity, and evaluated* in planta* for the control of* Verticillium* wilt and plant growth-promoting activity in eggplant. Evaluations of the antagonists were performed by the dual culture technique in agar plates. Separate biocontrol characteristics were further evaluated for their ability to protect eggplants against* Verticillium* wilt caused by* V. dahliae* and to promote eggplant growth in the greenhouse. The positive results obtained with* P. lilacinum* strain QLP12 deserve to be further investigated with a view to application under field conditions.

## 2. Material and Methods

### 2.1. Isolation of Fungal Strains Antagonistic to Different Fungi

Soil samples were collected from Qinling Mountain, Shaanxi Province, in China, and air-dried. A one-gram sample was added to 9 mL sterile water and cultivated in a shaker at 28°C for 1 h. The soil suspension was then serially diluted with sterile water with 50 *μ*L spread onto Potato Dextrose Agar (PDA) and incubated at 28°C for 5 days with purified and separated monospore strain. The colonies were streaked onto new PDA plates for purification. The purified colonies were coded and maintained on PDA medium and stored at 4°C.

The antagonism assay was conducted against different pathogenic fungi (*Botrytis cinerea*,* Fulvia fulva*,* Colletotrichum gloeosporioides*,* Mucor piriformis*,* Penicillium expansum*,* Trichothecium roseum*,* Rhizoctonia solani*,* Verticillium dahlia*,* Fusarium oxysporum* f. sp.* niveum,* and* F. oxysporum* f. sp.* vasinfectum*) by the dual culture technique. Ten indicator isolates were grown on PDA plates at 25°C for 5 days. A 5 mm diameter agar disk was placed on the center of a PDA plate and an equal amount of biocontrol strains was introduced on both sides of the same plate. All isolates were incubated on PDA plates at 25°C for 3–5 days. Then, inhibition zones were measured and inhibition rates were calculated, where IR = (*D*_CK_ − *R*_*T*_)/*D*_CK_, *D*_CK_, and *R*_*T*_ were the size of the inhibited area without and with biocontrol strains, respectively. This test was repeated twice and three plates for each treatment were used as replications. The strain with the strongest antifungal effect (inhibition zone) was selected for further studies. The strain QLP12 and pathogenic fungi growing on PDA or in Potato Dextrose Broth (PDB) at 28°C were stored long term in 20% glycerol at −20°C at the College of Plant Protection, Northwestern A&F University, Shaanxi, China.

### 2.2. Identification of the Strain QLP12 by Morphology and ITS rDNA Gene Sequence Analysis

Preliminary identification of the strain QLP12 was based on colony and mycelial morphology, including conidia production and type of conidiophores. The colony diameters were measured after the strain was incubated in darkness at 25°C for 7 days. The morphology of the cultured fungal strain was also observed by scanning electron microscope (SEM).

The strain QLP12 was further identified by ITS rDNA sequence analysis. Genomic DNA was extracted as described in earlier reports [11]. Universal primers ITS1-F and ITS4-R were used for amplification of the internal transcribed spacer (ITS) region [12]. One microliter of template was used in a 25 *μ*L amplification reaction which contained 12.5 *μ*L PCR Master Mix (Thermo Fisher Scientific), 1 *μ*L of each primer (20 *μ*M), and 10.5 *μ*L sterile distilled water. After initial denaturation at 95°C for 4 min, samples were cycled for 35 cycles using the following cycle profile: 95°C denaturation for 1 min, primer annealing at 55°C for 30 s, and primer extension at 72°C for 90 s, plus a further 10 min elongation step at 72°C. Amplified PCR products were separated by gel electrophoresis on 1.5% (w/v) agarose gel; they were then purified using a TIANgel Midi Purification Kit (Tiandz Technology, Ltd., Beijing, China) according to the manufacturer's instruction manual and sequenced by Genscript (Nanjing Biotech Co., Ltd., China). The ITS rDNA sequences of the strains were compared with available ITS rDNA sequences in GenBank databases using the BLAST search facility at the National Center for Biotechnology Information (NCBI). Sequences retrieved were aligned with the most similar-type strains obtained using CLUSTAL X [13]. Neighbor-joining (NJ) analysis and editing of the trees were performed using Mega 6.

### 2.3. Evaluation of the Growth-Promoting Effect of Strain QLP12 in Greenhouse Conditions

#### 2.3.1. Effects on Eggplant Seed Germination and Bud Growth

Eggplant cultivar Lanza number 1 was used in greenhouse and field experiments. After sterilization for 5 minutes in a 1% NaClO solution, eggplant seeds were washed three times in sterile distilled water (SDW) and air-dried under sterile conditions. The seeds were placed on filter paper in an 18 cm diameter Petri dish which had been soaked for 1 min in the fermentation broth (about 10^8^ cfu·mL^−1^) and incubated at 28°C for 6 d. Measurements of bud lengths and calculations of germination rates followed. The seeds soaked with liquid substrate were used as controls (the same below). Three Petri dishes were used for each treatment and each dish held ten seeds. This experiment was conducted three times.

#### 2.3.2. Colonization in the Rhizosphere and the inside of Eggplants

Separated eggplant seeds were sterilized as described above (Section 2.3.1) and seedlings at the three true-leaf stage were transplanted in sterile soil mixed with potting media (*m* :* m* = 2 : 1) and cultivated in the greenhouse at 25 ± 2°C, 70 ± 5% relativity humidity and 16 h light/8 h dark. Root, stem, leaf, and rhizosphere soil (1 g on their own) were sampled, each from 10 plants at 14, 19, 24, and 29 d. The colonization study depended on the tolerance of the strain QLP12 to distinctive fungicides. Using the dilution-plate method, the isolate was isolated from the PDA plate with 100 *μ*g·mL^−1^ 50% carbendazim WP and 50 *μ*g·mL^−1^ rifampicin. This procedure was followed by calculations of the colony or colonies. This experiment was conducted three times.

#### 2.3.3. Determination of Chlorophyll Content

In greenhouse, other eggplants were chosen to fulfill this task. An amount of 0.1 g tested plant material, sampled at 30 d after sowing, for example, leaves, was chopped into small pieces in a mortar and had 0.5 mL acetone, quartz sand, and 10 mL 80% acetone added and ground into a homogenate. The extract was poured into a 25 mL volumetric flask with 80% acetone. 80% acetone served as a blank test. Optical density (OD) was measured spectrophotometrically under 645, 552, 663, and 470 nm. According to the OD data, concentrations and contents of chlorophyll a, chlorophyll b, and total chlorophyll were calculated. This experiment was conducted three times, using ten plants for each treatment to collect these parameters.

#### 2.3.4. Determination of Root Vigor

For root activity tests, 0.5 g of root samples (Section 2.3.3) was placed into test tubes containing a 10 mL mixture of equal volumes of 0.4% 2,3,5-triphenyl tetrazolium chloride (TTC) and phosphate buffer, kept at 37°C for 1 h. Then 2 mL of 1 mol/L sulfuric acid was added to terminate the reaction. Careful grinding with 4 mL ethyl acetate and a small amount of quartz sand in a mortar to extract TTC followed. The OD of the extract was measured spectrophotometrically (SP-756PC) for 485 nm by colorimetry, taking the blank test as reference. In reference to the standard curve, the reducing amount of TTC was obtained. Based on the reduction amount of TTC, we could obtain a value for the reducing strength of the TTC. This experiment was replicated three times.

#### 2.3.5. Determination of Growth-Promoting Effect

When eggplants cultivated in the nursery with soil expanded their first true leaf, the plants were transplanted into pots with a 20 : 1 mixture of soil and fermentation broth of the QLP12 (about 10^8^ cfu·mL^−1^) diluted thereafter 100 times (10^6^ cfu·mL^−1^) and followed by a second treatment with QLP12 fermentation broth (10^6^ cfu·mL^−1^) 30 d after seeding transplantation. Mean plant height, number of leaves, leaf area (calculated by squares printed on coordinate paper), plant fresh height, root dry weight, and dry weight of leaves and shoot were recorded after 60 d and compared by ANOVA. This treatment that contained 10 pots was replicated 3 times with one plant per pot, using treatment with uncolonized substrate as controls. Experiments were analyzed independently.

### 2.4. Suppressive Effect of Strain QLP12 on* V. dahliae* in Greenhouse

The suppression of strain QLP12 against* V. dahliae* was examined under both greenhouse and field conditions to reveal its biocontrol efficiency on* Verticillium* wilt.

The strain QLP12 was cultured in PDB with 2% inoculum size at 28°C and 160 rpm for five days with a concentration of 10^8^ cfu·mL^−1^, being identical to that used with* V. dahliae* (10^8^ cfu·mL^−1^). Two treatments were carried out: (1)* V. dahliae *infested at the 3-leaf stage after treatment with QLP12 fermentation broth upon seeding, (2) treatments with both* V. dahliae *and QLP12 fermentation while transplanting, and (3) treatment with uncolonized substrate as controls. Each treatment, repeated three times, contained fifteen eggplants (cultivar “Lanza One”) planted individually in 15 nutrition pots (16 cm × 18 cm). The dosage of QLP12 and* V. dahliae* was 10 mL and 1 mL per plant, respectively.

According to the Agricultural Industry Standard of the People's Republic of China (NY/T 1464.34-2010), disease severity was evaluated about 40 days after pathogen infestation and was evaluated according to the following scales: 0, no diseased leaf; 1, <10%; 3, 11–25%; 5, 26–50%, 7, >50%; 9, plant killed. Disease index (DI) and control efficacy (CE) were calculated as follows:

(1) Disease index=∑number of stems with every scale×disease severity scorethe total number of leaves examined×the highest severity score×100,Control efficacy=the mean disease index of the control−the mean disease index of the treatmentthe mean disease index of the control×100%.

### 2.5. Suppressive Effect of QLP12 on* V. dahliae* in the Field

Further field experiments using the same eggplant cultivar to determine the biocontrol efficacy of QLP12 on* V. dahliae* were conducted in Yangling District, Shaanxi Province, China. The size of each plot was 2 × 5 m and about 60 eggplants were grown in each plot. Except for the different treatments, the field and crop were managed using the same practices as the eggplant production in this region. A completely random design was used with three replications per treatment for each experiment. QLP12 and* V. dahliae* were obtained as described (Section 2.4) with a concentration of 10^8^ cfu·mL^−1^. One liter of QLP12 fermentation broth was poured into 1 m^2^ soil at the time of sowing. 0.1 L* V. dahliae* was inoculated on eggplants at the 3-leaf stage by pouring on roots. The experiment consisted of three treatments: (1) artificial pathogenic fungus infested but treated with QLP12 fermentation broth; (2) artificial pathogenic fungus infested but challenged with fungicide, 1000-fold dilution of 50% carbendazim WP; (3) uncolonized substrate treatment as the control.

Disease severity, described in Section 2.4, was evaluated every 20 days after infestation with the pathogen 30 days thereafter.

### 2.6. Statistical Analysis

Calculations and comparisons of treatment means for each experiment were conducted using analysis of variance (ANOVA) and the SPSS 20.0 software; means were separated by Tukey Honestly Significance Difference (Tukey HSD) test at *P* = 0.05.

## 3. Results

### 3.1. Inhibition Activity of Strain QLP12* In Vitro*

Through the result of dual culturing on PDA (Table 1), QLP12 showed a strong ability (IR ≥ 65%) to inhibit mycelium growth in several selected phytopathogenic fungi, such as* M*.* piriformis*,* T*.* roseum*,* R*.* solani*, and* V. dahliae*. Inhibitory effects (77.7%) against* M*.* piriformis *were most pronounced. A lower activity (40% to 65% inhibition rates) was calculated against* C. gloeosporioides *and* F. fulva*. Mycelial growth in the remaining fungi was inhibited to some extent.

### 3.2. Identification of Strain QLP12

**Figure 1:**
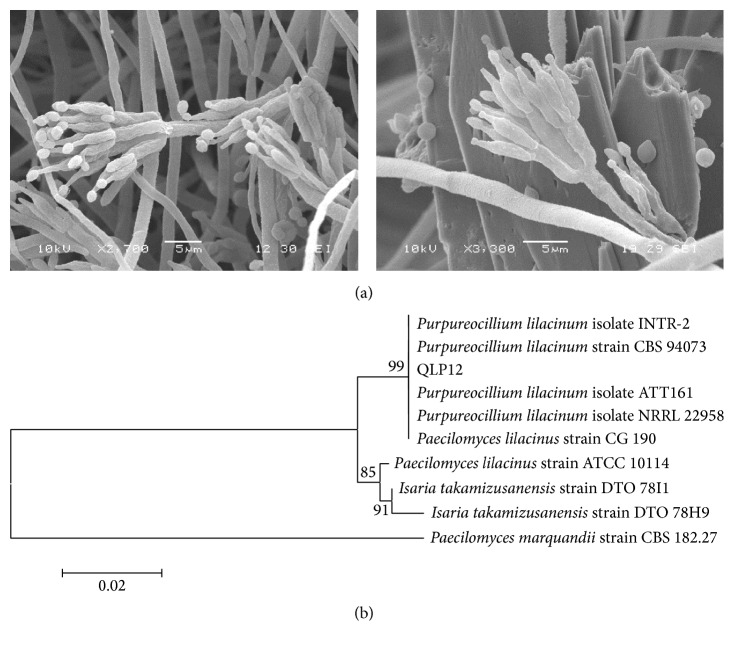
(a) SEM photographs of* Purpureocillium lilacinum* strain QLP12. (b) Phylogenetic tree for* P. lilacinum* and its related species based on full-length ITS rDNA sequences constructed using the neighbor-joining method. Note: the number at each branch refers to the percentages of times the group of strains in that branch occurred, based on 1000 cycles in bootstrap analysis.

The strain QLP12 was identified by morphological examination and molecular study. The fungi are largely characterized on the basis of their morphological characters (Figure 1(a)). The macroscopic characters of a fungus growing on agar can provide useful and rapid clues to identify its respective genus. The diameter of a QLP12 colony cultured for 7 days on CMA at 25°C was 2.7~3.8 cm with felty structure and aerial mycelium. Figure 1(a) shows that the lengths of the conidiophores varied widely (15.6~43.5 *μ*m × 2.5~3.5 *μ*m), had swollen bases, and displayed 1~2 *μ*m long necks on their top ends. The conidiophore presents a broom branch, and the conidia themselves were catenulate and oval, 2.5~3.75 *μ*m × 2.5~3.0 *μ*m without forming chlamydospores.

The ITS sequences aligned in the GenBank database revealed that strain QLP12 matched closest with* Purpureocillium lilacinum*. In the phylogenetic tree (Figure 1(b)), the organism most similar to the strain QLP12 according to the results of alignment was* P. lilacinum*. Based on the features shown by morphology and molecular biology, the strain QLP12 was identified as* P*.* lilacinum*.

### 3.3. Colonization of QLP12 in the Rhizosphere and inside of Eggplants

**Figure 2:**
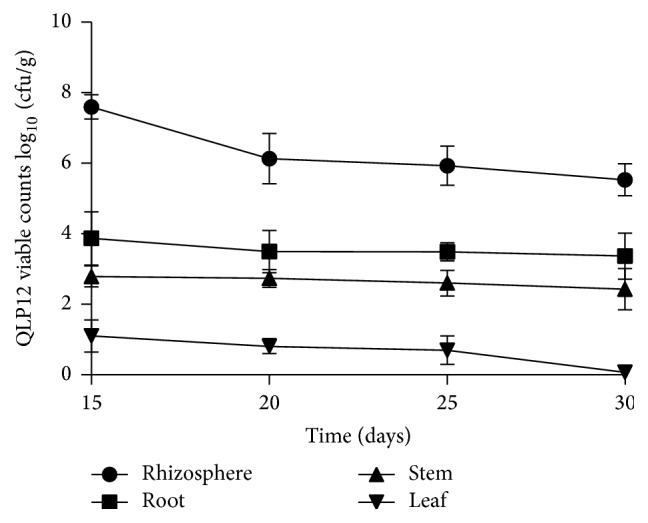
The colonization kinesis of QLP12 on and/or in eggplant.

To examine the ability of QLP12 to colonize germinating eggplant seedlings, incubated eggplant seeds were planted in QLP12-amended sterile soils with a 16 h photoperiod. The result showed that QLP12 can stably colonize in the rhizosphere and enter plants with the highest biomass (7.6 × 10^4^ cfu·g^−1^) (Figure 2). A population of QLP12 isolates showed a stable trend with extended time, still receiving about 10^4^ cfu·g^−1^ after 30 days. The result of the respective colonization from high to low in root, stem, and leaf agreed with the distribution and transmission regularities of microbes in plants.

### 3.4. Growth-Promoting Effect of QLP12 under Greenhouse Conditions

Various concentrations of QLP12 fermentation promoted eggplant seed germination and bud growth and stimulated the growth of the seedling. The growth-promoting effect of QLP12 is excellent when fermentation broth is diluted to 10^6^ cfu·mL^−1^ (Table 2). Eggplant bud growth showed an improvement in both bud length and germination rate, which was 12.2 mm and 76.7%, respectively, compared with 6.8 mm and 60.2% for the control.

Through greenhouse experiments, using QLP12 fermentation broth with different effects on eggplant growth revealed significant growth promotion ability to affect fresh and dry weights and achieving growing rates of 132.1% (aerial part) and 110.0% (root) (Table 3). QLP12 gave eggplants vigorous growth and greener leaves based on morphologic observation. It is speculated that QLP12 causes the release of some secretions that can promote the growth of plants to this observed huge extent in plant dry weight.

Chlorophyll content and root activity were further identified as possible physiological reasons for the growth promotion effect of strain QLP12 as the effects of QLP12 on chlorophyll content and root activity were significant (Table 4). Compared with the control, eggplants treated with QLP12 showed that total chlorophyll content in the leaf and root activity had increased 47.83% and 182.02%, respectively.

### 3.5. Greenhouse Suppression on* Verticillium* Wilt

The results of pot experiments in the greenhouse (Table 5) indicated that QLP12 fermentation broth can suppress* Verticillium* wilt on eggplant. Pretreatment of the soil with QLP12 fermentation broth can dramatically decrease the rate of disease development in the plants (43.6%), DI (83.8%), and control the effects of the disease. Although inoculating pathogenic fungus and QLP12 together did not reduce the incidence of the disease, it obviously decreased the disease index and showed a better control efficiency. Through all the three measurable targets, pouring QLP12 fermentation broth into the soil in advance showed a better suppressive effect which diminished 43.6% and 68.7% of the disease rate and DI, respectively, and enhanced 42.2% of the control effects.

### 3.6. Field Suppression on* Verticillium* Wilt

**Figure 3:**
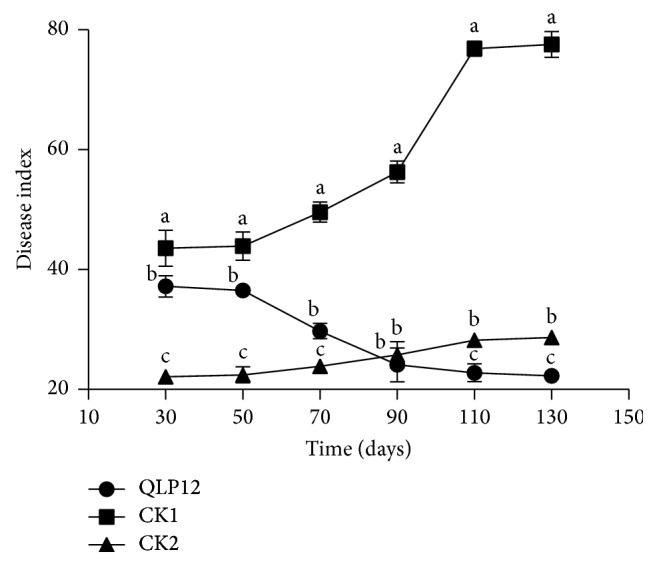
The dynamic variation of eggplant* Verticillium* wilt disease index under different soil treatments. Note: three types of data in the same day followed by different lower cases indicate significant differences at the *P* = 0.05 level of confidence. CK1 and CK2 mean the treatment with water and carbendazim, respectively.

The results indicate that the application of QLP12 fermentation broth significantly suppressed* Verticillium* wilt (Figure 3). There was a stable trend in the rise of the control (CK1) and the carbendazim treatment (CK2), from 43.6 of the DI to 77.6 and 22.1 to 28.7, respectively, throughout the whole 130 days. By contrast, unlike CK1 and CK2, the DI of QLP12 decreased during the same period, the rate reaching 40.1%. The disease index showed a sharp increase, from a beginning on the 50th day to an end on the 110th day. DI of the control and carbendazim kept increasing, while the decline of DI of QLP12 was much in evidence. As time went on, the DI of QLP12 was slightly below carbendazim at around the 90th day and significantly (*P* < 0.05) decreased by 110th day with a minimum value occurring on 130th day (DI = 22.3).

## 4. Discussion

Keeping a balanced plant pathosystem with beneficial soil microorganisms to suppress soil-borne disease such as wilt by* V. dahliae *biologically and increase plant tolerance to disease is the goal of disease management by biocontrol in sustainable production systems for crops. Pollution from intensive farming caused by the indiscriminate use of fungicides and fertilization made a mess of the soil microenvironment, facilitating outbreaks of plant diseases. This paper demonstrated that the strain QLP12 identified as* P. lilacinum* reduced the DI of eggplant* Verticillium* wilt and promoted plant growth via physiological and biochemical routes.

Current intensive farming practices can lead plants to lose resistance to diseases, the latter becoming more and more serious and widespread. Using fungicides for chemical control is one major measure of plant disease control including that for eggplant* Verticillium* wilt. Short-lasting effects, however, affect and restrict the use of fungicides. Furthermore, traditional chemical controls cannot be used due to high residue or in pollution-free production systems where the use of synthetic pesticides is not allowed [14]. The biological control of plant diseases, on the other hand, represents an environmentally friendly alternative, which uses profitable microorganisms to lead to the arrest of pathogen growth and sometimes even promotes plant growth. Biocontrol methods have been widely explored over the past years and they depend mainly on the selection of the appropriate BCAs to inhibit plant pathogen growth, preventing disease or decreasing damage, in an environmentally friendly way.

BCAs can show a long-lasting effect to suppress pathogens as long as they remain established and develop in the rhizosphere or within plants. In our greenhouse experiments, QLP12 exhibited strong suppressive activity and stable development in the rhizosphere, colonizing eggplants with ease. Using scanning electron microscopy (SEM), Cavello et al. [15] revealed that the colonization of* P. lilacinum *occurs on the root hair surface. We also found that QLP12 is capable of colonizing tomato, potato, wheat, and cucumber, as well as the surface of apple fruits and leaves (data not shown).

Chlorophyll, a vitally important molecule in photosynthesis, helps plants to transform the energy content of light to energy needed by the plant itself. By converting solar energy into chemical energy, plants grow stronger with a higher concentration of chlorophyll. Root vigor is one of the indicators which mirror the ability of water and nutrient uptake, as well as synthesis and storage of metabolites by the plant. A plant growth-promoting effect on height, leaf area, fresh weight, and so forth was revealed and probably increases of chlorophyll concentration in combination with an improvement of root vigor were the reason. Various biocontrol agents possess properties to promote plant growth, leading to stronger and faster growing plants.

*Purpureocillium lilacinum* has been mainly considered as a parasitic fungus on eggs, female adults, and larvae of insects and especially nematodes [16–20]. Liu et al. [18] found a 15% synergistic effect on the control of root-knot nematode by* P. lilacinum* YES-2 combined with Fosthiazate in a 5-year glasshouse experiments. Application of* P. lilacinus* in pot experiments by [21] provided effective control of second-stage juveniles, eggs, or egg masses of root-knot nematodes. In further greenhouse experiments, the* P. lilacinum *was exposed potential control of root-knot nematodes either as a stand-alone method or in combination with soil solarization. Lopez and Sword [22] treated cotton seeds with* P. lilacinum* conidia which resulted in significant increases in plant dry biomass, plant developmental stage, and number of squares. Four* P. lilacinum* strains were identified by Sabet et al. [23], which showed a maximum 65% nematode control under greenhouse condition, indicating an excellent potential in reducing nematode population. Apart from its pathogenicity to insects,* P. lilacinum* is recognized as an entomopathogenic fungus and possesses a plant growth promotion ability for various crops, that is, tomato [20, 24, 25], mung bean [26], and banana [27]. Similarly, Lopez and Sword [22] reported that* P. lilacinum* showed a convincing effect on promoting cotton growth, leading to significantly larger plant sizes. Some research focused on a combination of* P. lilacinum* and organic fertilizer that resulted in considerable increases in the plants [28, 29]. This powerful growth vigor can lead to a greater tolerance towards pathogens and needs to be seen as a momentous achievement in biological control. However, there is little research that focuses on the biocontrol of plant diseases, especially eggplant* Verticillium* wilt by* V. dahliae*. The aim of our study on the biocontrol of plant diseases is to apply BCAs in the field. In this paper, QLP12 showed a noteworthy ability to control eggplant* Verticillium* wilt with a minimum DI, which was significantly lower than that of carbendazim. Although the microbial biomass decreased to 10^6^ cfu·g^−1^ thirty days after application of QLP12 fermentation broth, it still, successfully, in the field experiment, suppressed the eggplant* Verticillium* wilt in the next 100 d and showed an effective concentration which maintained the biological control to this disease. It is worth noting that both QLP12 and carbendazim can decrease the DI of wilt disease, but they showed contrary variation tendencies as time went on. Eggplants treated by QLP12 fermentation broth exhibited a decline in DI with larger variation amplitude from days 50 to 90. By contrast, DI increased in the carbendazim treatment with a gradual decrement control effect. This result revealed a suitable example of long-duration efficacy by BCAs and a short one by chemical fungicides.

BCAs possess high selectivity to target pathogens leading to direct suppression. Strain QLP12 showed especially strong ability to inhibit* V. dahliae*,* M*.* piriformis,* and* T*.* roseum *but had less influence on others. Meanwhile, with the inherent advantage of restoring soil microecology, adding BCAs into the soil can protect soil to harden. Pouring strain QLP12 fermentation broth onto the root system of 3-leaf stage seedlings effectively gave the agriculturist a way to control eggplant* Verticillium* wilt. However, it remains unclear how* P*.* lilacinum *QLP12 suppresses the incidence and severity of leaf wilt caused by* V. dahliae* on eggplant. Further research will have to involve other crops to explore the growth-promoting ability, followed by a study on the mechanisms of secretase, resistance signal transduction, and so on. Numerous BCAs without suppressive effects in the field have limitations, but strain QLP12 keeps suppression of* V. dahliae* stable and effective and moreover promotes eggplant growth indoors and outdoors. It therefore deserves a deeper look into its long term effectiveness, which will require further research.

## Figures and Tables

**Table 1:** Inhibitory effects of the strain QLP12 on 10 target pathogens.

	Pathogens									
	*M. p*	*T. r*	*C. g*	*B. c*	*R. s*	*P. e*	*V. d*	*F. o v*	*F. o n*	*F. f*
Inhibition rate (%)	77.7 ± 1.26^a^	66.6 ± 1.72^b^	47.1 ± 2.93^d^	36.7 ± 2.51^e^	66.7 ± 1.83^b^	38.9 ± 1.41^e^	66.7 ± 1.00^b^	36.8 ± 2.11^e^	62.5 ± 2.16^c^	46.2 ± 1.50^d^

Note: data (mean ± SD) in the table are the averages of three replicates. Data followed by different lower case show statistically significant differences at *P* < 0.05 according to Tukey's HSD.

**Table 2:** The effect of strain QLP12 on seed germination and bud growth of eggplant with different concentrations of fermentation broth.

Concentration of fermentation broth	Bud length (mm)	Germination rate (%)
10x	6.1 ± 0.6^b^	62.8 ± 3.9^bc^
50x	10.8 ± 0.4^a^	69.9 ± 7.6^ab^
100x (10^6^ cfu·mL^−1^)	12.2 ± 1.7^a^	76.7 ± 2.4^a^
CK	6.8 ± 0.4^b^	60.2 ± 3.4^c^

Note: data (mean ± SD) in the table are the averages of three replicates. Data followed by different lower case show statistically significant differences at *P* < 0.05 according to Tukey's HSD.

**Table 3:** The effect of QLP12 fermentation broth on growth and development of eggplant.

	Height of plant/cm	Leaf area/mm^2^	Fresh weight/g	Dry weigh of overground parts/g	Dry weigh of roots/g
QLP12	41.7 ± 1.76^*∗*^	254.68 ± 1.80^*∗*^	36.66 ± 1.24^*∗*^	3.23 ± 0.10^*∗*^	2.18 ± 0.12^*∗*^
CK	32.7 ± 0.87	173.78 ± 1.83	15.79 ± 1.61	1.54 ± 0.16	1.34 ± 0.05

Note: the asterisk indicates a significant difference at *P* < 0.05.

**Table 4:** The effect of QLP12 fermentation broth on chlorophyll content and reduction intensity of eggplant.

	C_a_ (mg/L)	C_b_ (mg/L)	C_T_ (mg/L)	Reduction intensity (*μ*g·g^−1^·h^−1^)
QLP12	3.321 ± 0.108^*∗*^	2.816 ± 0.115^*∗*^	6.194 ± 0.100^*∗*^	0.243 ± 0.004^*∗*^
CK	2.249 ± 0.080	2.219 ± 0.131	4.225 ± 0.017	0.102 ± 0.007

Note: C_a_, C_b_, and C_T_ mean chlorophyll a, chlorophyll b, and total chlorophyll, respectively.

**Table 5:** Control of eggplant *Verticillium* wilt in greenhouse by strain QLP12.

Treatments	Rate of diseased eggplant (%)	Disease index	Control effects (%)
T1	56.4 ± 1.6^b^	14.03 ± 1.80^c^	83.82 ± 2.23^a^
T2	100.0 ± 0.0^a^	44.77 ± 4.12^b^	48.42 ± 5.27^b^
CK	100.0 ± 0.0^a^	86.87 ± 2.87^a^	

Note: T1 means strain QLP12 was treated before *V*.* dahlia*; T2 means strain QLP12 and* V*.* dahlia *were treated at the same time; CK means the treatment of *V*.* dahlia* only. Data (mean ± SD) in the same volume are the averages of three replicates. Data followed by different lower case show statistically significant differences at *P* < 0.05 according to Tukey's HSD.

## References

[1] Klosterman S. J., Atallah Z. K., Vallad G. E., Subbarao K. V. (2009). Diversity, pathogenicity, and management of *Verticillium* species. *Annual Review of Phytopathology*.

[2] Pegg G. F., Brady B. L. (2002). Control. *Verticillium Wilts*.

[3] Papasotiriou F. G., Varypatakis K. G., Christofi N., Tjamos S. E., Paplomatas E. J. (2013). Olive mill wastes: a source of resistance for plants against *Verticillium dahliae* and a reservoir of biocontrol agents. *Biological Control*.

[4] Hu X., Roberts D. P., Xie L. (2013). *Bacillus megaterium* A6 suppresses *Sclerotinia sclerotiorum* on oilseed rape in the field and promotes oilseed rape growth. *Crop Protection*.

[5] Fahima T., Henis Y. (1995). Quantitative assessment of the interaction between the antagonistic fungus *Talaromyces flavus* and the wilt pathogen *Verticillium dahliae* on eggplant roots. *Plant and Soil*.

[6] Marois J. J., Johnston S. A., Dunn M. T., Papavizas G. C. (1982). Biological control of Verticillium wilt of eggplant in the field. *Plant Disease*.

[7] Bubici G., Marsico A. D., D'Amico M., Amenduni M., Cirulli M. (2013). Evaluation of *Streptomyces* spp. for the biological control of corky root of tomato and Verticillium wilt of eggplant. *Applied Soil Ecology*.

[8] Tyvaert L., França S. C., Debode J., Höfte M. (2014). The endophyte Verticillium Vt305 protects cauliflower against Verticillium wilt. *Journal of Applied Microbiology*.

[9] Gizi D., Stringlis I. A., Tjamos S. E., Paplomatas E. J. (2011). Seedling vaccination by stem injecting a conidial suspension of F2, a non-pathogenic *Fusarium oxysporum* strain, suppresses Verticillium wilt of eggplant. *Biological Control*.

[10] Li J.-G., Jiang Z.-Q., Xu L.-P., Sun F.-F., Guo J.-H. (2008). Characterization of chitinase secreted by *Bacillus cereus* strain CH2 and evaluation of its efficacy against Verticillium wilt of eggplant. *BioControl*.

[11] Deng J. X., Paul N. C., Li M. J., Seo E. Y., Sung G. H., Yu S. H. (2011). Molecular characterization and morphology of two endophytic *Peyronellaea* species from *Pinus koraiensis* in Korea. *Mycobiology*.

[12] Gardes M., Bruns T. D. (1993). ITS primers with enhanced specificity for basidiomycetes—application to the identification of mycorrhizae and rusts. *Molecular Ecology*.

[13] Thompson J. D., Gibson T. J., Plewniak F., Jeanmougin F., Higgins D. G. (1997). The CLUSTAL X windows interface: flexible strategies for multiple sequence alignment aided by quality analysis tools. *Nucleic Acids Research*.

[14] Ongena M., Jacques P. (2008). *Bacillus* lipopeptides: versatile weapons for plant disease biocontrol. *Trends in Microbiology*.

[15] Cavello I. A., Hours R. A., Rojas N. L., Cavalitto S. F. (2013). Purification and characterization of a keratinolytic serine protease from *Purpureocillium lilacinum* LPS # 876. *Process Biochemistry*.

[16] Barra P., Etcheverry M., Nesci A. (2015). Improvement of the insecticidal capacity of two *Purpureocillium lilacinum* strains against *Tribolium confusum*. *Insects*.

[17] Hotaka D., Amnuaykanjanasin A., Chan M., Siritutsoontorn S., Maketon M. (2015). Efficacy of *Purpureocillium lilacinum* CKPL-053 in controlling Thrips palmi (*Thysanoptera: Thripidae*) in orchid farms in Thailand. *Applied Entomology & Zoology*.

[18] Liu J., Sun J., Qiu J., Liu X., Xiang M. (2014). Integrated management of root-knot nematodes on tomato in glasshouse production using nematicides and a biocontrol agent, and their effect on soil microbial communities. *Nematology*.

[19] Schapovaloff M. E., Alves L. F. A., Fanti A. L., Alzogaray R. A., Lastra C. C. L. (2014). Susceptibility of adults of the cerambycid beetle *Hedypathes betulinus* to the entomopathogenic fungi *Beauveria bassiana, Metarhizium anisopliae*, and *Purpureocillium lilacinum*. *Journal of Insect Science*.

[20] Singh S., Pandey R. K., Goswami B. K. (2013). Bio-control activity of *Purpureocillium lilacinum* strains in managing root-knot disease of tomato caused by *Meloidogyne incognita*. *Biocontrol Science and Technology*.

[21] Anastasiadis I. A., Giannakou I. O., Prophetou-Athanasiadou D. A., Gowen S. R. (2008). The combined effect of the application of a biocontrol agent *Paecilomyces lilacinus*, with various practices for the control of root-knot nematodes. *Crop Protection*.

[22] Lopez D. C., Sword G. A. (2015). The endophytic fungal entomopathogens *Beauveria bassiana* and *Purpureocillium lilacinum* enhance the growth of cultivated cotton (*Gossypium hirsutum*) and negatively affect survival of the cotton bollworm (*Helicoverpa zea*). *Biological Control*.

[23] Sabet O. M., Sharifnabi B., Tehrani A. A. (2013). Biological control of the root-knot nematode, *Meloidogyne javanica* by four isolates of *Paecilomyces lilacinus* and an isolate of Isaria farinosa on tomato plants. *Iranian Journal of Plant Pathology*.

[24] Munawar M., Khan S. A., Javed N., Ul Haq I., Gondal A. S. (2015). Bio-management of tomato wilt complex caused by *Meloidogyne incognita* and *Fusarium oxysporum* f. sp. *lycopersici*. *Nematology*.

[25] Oclarit E. L., Cumagun C. J. R. (2009). Evaluation of efficacy of *Paecilomyces lilacinus* as biological control agent of meloidogyne incognita attacking tomato. *Journal of Plant Protection Research*.

[26] Mansoor F., Sultana V., Ehteshamul-Haque S. (2007). Enhancement of biocontrol potential of *Pseudomonas aeruginosa* and *Paecilomyces lilacinus* against root rot of mungbean by a medicinal plant *Launaea nudicaulis* L. *Pakistan Journal of Botany*.

[27] Wang J., Wang G. F., Yang L. Y. (2013). Effects of *Paecilomyces lilacinus* application and intercropping on controlling Fusarium wilt of banana. *Journal of Fruit Science*.

[28] Liu H. Q., Di W. U., Zhang A. X., Hai-Lian M. A., Shi-Dong L. I., Guo R. J. (2012). Influence of adding *Paecilomyces lilacinus* to biological organic fertilizer on growth of cucumber in warmhouse. *Journal of Hebei North University*.

[29] Siddiqui Z. A., Futai K. (2009). Biocontrol of *Meloidogyne incognita* on tomato using antagonistic fungi, plant-growth-promoting rhizobacteria and cattle manure. *Pest Management Science*.

